# Assessment of Households' Willingness to Join and Pay for Improving Waste Management Practices in Gedeo Zone, Southern Ethiopia

**DOI:** 10.1155/2022/9904665

**Published:** 2022-08-04

**Authors:** Abdene Weya Kaso, Habtamu Endashaw Hareru, Zemachu Ashuro, Negasa Eshete Soboksa

**Affiliations:** ^1^School of Public Health, College of Medicine and Health Science, Dilla University, Ethiopia; ^2^Department of Environmental Health, School of Public Health, College of Medicine and Health Science, Dilla University, Ethiopia

## Abstract

**Background:**

Waste management has become a serious challenge in urban areas of developing countries. However, managing municipal solid waste generated is the most costly urban service and needs community engagement in management of municipal solid wastes. Therefore, this study determined willingness to join and pay for improving solid waste management services and associated factors among households of Gedeo zone, Southern Ethiopia.

**Method:**

We performed a community-based cross-sectional study design to assess willingness to join and pay for improved solid waste management and its predictors among residents in Gedeo zone, Southern Ethiopia. Multivariate logistic regression analysis was computed to identify the factors associated with willingness to join and pay for improved solid waste management services. An adjusted odds ratio (AOR) with a 95% confidence interval (CI) was used to report an association, and a *p* < 0.05 was used to declare a statistically significant association.

**Result:**

Of 552 study participants, 91.5% (95% CI: 89.2, 93.8) were willing to join and 86.3% (95% CI: 83.3, 89.4) of them were willing to pay for improving solid waste management services. Having a large family size, higher educational status, positive attitude, and good knowledge of waste management services, access to waste management services, and amount of waste generated per week were positively associated with willingness to support improved solid waste management services. In addition, we found that respondents with high educational status, monthly income, who had good knowledge of waste management, access to waste management services, and the amount of waste generated per week were significant predictors of willingness to pay for improved solid waste management.

**Conclusion:**

This study found that a significant number of the respondents were willing to support and pay for improved solid waste management services. A higher probability of willingness to support and pay for solid waste management services among residents who had access to waste management services and good knowledge of solid waste management was observed. Therefore, wide-range awareness creation through mini media should be used to address poor knowledge. In addition, establishing more temporary waste collection sites in every corner of cities is needed to encourage households to support and pay for improved solid waste management services.

## 1. Introduction

Waste management has become a serious challenge in urban areas of developing countries. It is a global environmental and public health problem and threatened the lives of millions of communities living in urban areas [1–3]. As a result of rural to urban migration, industrialization, urbanization, and rapid population growth, municipality waste generation is expected to rise to 2.2 billion tons by 2025 [4, 5]. Every year, the world's cities generate 1.3 billion tons of waste annually, which is expected to increase from 2 billion to 3.4 billion metric tons by 2050. Poor waste management increases the risk of disease spread and the contamination of water sources. As a result of improper waste management, between 400,000 and 1 million deaths may occur annually in developing countries [6–8].

By 2030, Africa is expected to have an urban population of over 50% and an urban growth rate of 3.4% [9]. Though rapid urbanization and population growth have opportunities and benefits in terms of economies and social activities, it brings enormous challenges to improving waste management services. This has an indispensable impact on socioeconomic and environmental sides in cities of Africa [5]. In 2015, African countries have generated around 200 thousand or more tons of solid waste per day [10]. However, managing municipal solid waste generated is the most costly urban service and absorbs 20-40% of municipal revenues in developing countries [11]. As a result, most African countries suffer from an imbalance between the amount of waste generated and the capacity to manage their wastes properly [12–14]. For example, in Egypt, approximately 10 to15 million tons of solid waste is generated annually, with around 15%-65% of waste being collected and transported efficiently [7]. In addition, in Kampala, out of 1600 tons of solid wastes generated per day, 45-50% of the tones remained uncollected [15], while 42.5% of 97.092 kg/day of wastes generated from households in Ethiopia were dumped on the roadsides and open fields [16]. In addition, in Dilla town, the average per capita waste generation rate of residential households was estimated to be 0.475 kg/capita/day, with improper collection and transportation [17]. Unfortunately, the dumping of solid wastes into open spaces or drainages causes overflooding and environmental pollution in the cities [3, 18]. Thus, establishing effective and sustainable municipal sanitation in emerging cities is crucial to halt public health and the environmental problems caused by poor solid waste management [19]. However, as the provision of environmental sanitation services needs huge resources, the amount of money spent on these services depends on resources mobilized from residents or the economic status of the countries [13, 20].

According to the World Bank, high-income countries spend around $100 per tons on waste management services, whereas the developing countries' expenditure was $35 per ton [8]. In developing countries, decision-makers were mainly confronted with rapid urbanization and problems of dysfunctional environmental sanitation facilities and services due to insufficient resource mobilization. Thus, the provision of effective waste management services needs the engagement of the private sector, local communities, and developmental partners [8, 15]. However, the preference of communities to support and afford for management of wastes produced varies from country to country. A previous study done in Nepal indicated that 61% of households were willing to pay 0.72 US$ per month for waste management [18]. Besides, in Nigeria, 80% of the households were in support of residential waste management [21], whereas 48.1% of households in Uganda were willing to pay USD 2.9 monthly for improving solid waste management (SWM) services [22]. In the Ethiopian context, 78% of residents in Addis Ababa [23], 86.3% in Bahir Dar city [24], 83.5% in Jimma town [2], and 81.06% in Injibara town [25] were willing to support or pay for SWM services. However, households' willingness to support and pay for solid waste management services depends on different sociodemographic, economic, attitude, and awareness-related factors. Age, income, educational status, sex, awareness of the household towards the hazard related to waste produced, possession of the house, and the amount of waste generated have been identified in previous studies as determinants of willingness to pay (WTP) [2, 18–20, 23–28].

Despite the significance of environmental economic valuation studies for the integrated management and decision-making process regarding environmental resources, only limited studies that focused mainly on willingness to pay were done in Ethiopia. In addition, previous studies conducted in the Gedeo zone found unavailability of collection vehicles, inappropriate setting of waste containers, and ineffective solid waste fee system as the barrier to SWM [17]. However, to avert the potential risks to the environment and human health caused by air and water pollution, there is a need for effective, efficient, and sustainable management of urban solid wastes and overcoming barriers to SWM [29]. Thus, to bring an improvement in the SWM service provided in terms of quality and timely accessibility, a reformation of the solid waste fee system is needed to meet the changing healthcare markets. Therefore, this study determined the household willingness to join and pay for improved solid waste management in the Gedeo zone, Southern Ethiopia.

## 2. Method and Material

### 2.1. Study Setting, Design, and Period

We performed a community-based cross-sectional study among households of the Gedeo zone, Southern Ethiopia from 10 March to 5 April 2022. Gedeo zone is located in the Southern Nations, Nationalities, and Peoples Region (SNNPR) and is surrounded by Sidama Region and Oromia Region. According to information from Gedeo zonal health office data, it has six districts and two city administrations, with a total population of 1,247,812 (624,931 men and 622,881 women). The zonal town, Dilla town, is located 365 kilometres from Addis Ababa and 90 kilometres from Hawassa, the capital of SNNPR. The average per capita waste generation rate in this town from residential households was estimated to be 0.475 kg/capita/day. The majority (68.4%) of solid waste generated by residents was organic which can be used as organic fertilizer in urban agriculture. However, improper solid waste collection, disposal, and transportation were a huge problem due to an ineffective waste fee system, lack of trained manpower, and unavailability of collection vehicles [17].

### 2.2. Study Population and Eligibility Criteria

The source population was all households in the selected town, while all households in the selected kebeles of the selected town were the study population. Households' heads older than 18 years and who reside for 6 months in the selected kebeles were included in the study. Critically ill household heads that were not able to respond for the interviews were excluded.

### 2.3. Sample Size Determination and Sampling Technique Procedure

We calculated a sample size of 581 by a single population proportion formula, taking the proportion of willingness to support improved waste management services, 78% [23], 95% confidence level, 5% margin of error, design effect of 2, and 10% nonresponse rate. A two-stage sampling technique was employed to select participating households. First, two town administrations (i.e., Dilla town and Yirga Chaffe town) within the Gedeo zone were selected purposively based on the community-based team training program finding on the status of solid waste management in these towns. Secondly, six kebeles (three from each town) were randomly selected using a lottery method, and the sample was proportionally allocated to each kebele (the smallest administrative unit) based on the size of residents in each kebele. Finally, a systematic random sampling method was used to access the participants.

### 2.4. Variables of the Study

Dependent variables were the level of willingness to join and pay for improved solid waste management services among residents of the Gedeo zone. Sociodemographic characteristics (age, sex, educational status, family size, and occupational status), economic factors (monthly income and house ownership), service-related factors (quantity of waste generated, access to waste management services, and household satisfaction towards current services), and perception-related factors (awareness and attitude about waste management services) were independent variables of the study.

### 2.5. Operational Definitions

Waste management: a process of managing residential solid wastes produced by households and enterprises as a process of consumption

Willingness to pay: defined as the households' preference to pay a specified amount of money for improved solid waste management, elicited using a double bounded contingent valuation method (CVM) specifically by applying a bidding game. The CVM is a type of stated preference approach used to estimate the WTP for many healthcare services and has become the most commonly used valuation technique because of its flexibility and its ability to determine total values

Willingness to join: defined as the household preference to support the provision or improvement of solid waste management in their area, determined by close-ended binary questions

Knowledge towards waste management: we assessed the households' knowledge of solid waste management using 14 yes or no items measuring knowledge of waste management methods and the impact of inappropriately managed wastes on health, environment, and livestock. We assigned a value of “1” for correct response (yes) and “0” for incorrect response (no). Then, we computed the knowledge score and converted it to a percentage, in which the respondents who had scored 60% and above were considered as good and poor otherwise

### 2.6. Attitude towards Waste Management

We measured the household attitudes towards solid waste management by six questions on Likert's scales ranging from strongly disagree to strongly agree. The total attitude score ranged from 6 to 30, with a score of 18 and above denoting a “positive attitude” and those who scored below 18 points were considered to have a “negative attitude” towards waste management services. The internal reliability of attitude was assessed using Cronbach *α* and found the Cronbach *α* of 0.82, indicating internal reliability.

### 2.7. Data Collection Procedures and Quality Management

We used a semistructured, interviewer-administered questionnaire to collect data from households. We adapted this questionnaire from previous studies conducted on WTP for improved SWM for data collection purposes [25, 26, 28, 30]. We prepared the questionnaire initially in English and then translated it into Amharic and Gedeu'ffa (the local language) and later on back to English to check its consistency. We performed a pretest on 5% of the sample in Wenago town, with almost similar characteristics to our study areas, and six health officer graduates collected data after two days of training on data collection. Supervisors supervised the data collectors and provided any necessary correction on the spot, while the principal investigator checked the completeness of the data on daily basis. We assessed households' willingness to join waste management using yes or no questions. Accordingly, if the individuals responded as “yes,” we considered them as “willing to join” and asked them for their WTP for improved solid waste management services. However, if the respondent responded as “no,” we considered them as “not willing to join” and they were asked for the reasons why they refused to support solid waste management services. Before assessing households' willingness to pay, we conducted a pilot study to determine initial bid values. The responses of WTP from the pilot study were summed to provide the average initial bid of 30 Ethiopian birr (ETB) per month. We measured the WTP for improved solid waste management services using the dichotomous choice CVM by asking a specific amount of money (30 ETB) and probed the question using a higher or lower bid value depending on the individuals' response until the maximum amount of contributions was achieved.

### 2.8. Data Analysis

We entered data into Epi-Info 7 and exported it to Statistical Package for Social Science (SPSS) version 25 for analysis. We used descriptive statistics such as mean with standard deviation (SD) for continuous variables, and frequency and the percentage were utilized to summarize for categorize variables. Multivariate logistic regression analysis was computed to identify the factors associated with willingness to join and pay for improving solid waste management services. All variables with a *p* value less than or equal to 0.25 in the bivariate logistic regression analysis were transferred into multivariate logistic analysis and a *p* value of less than or equal to 0.05 with an AOR with 95% CI was used to declare the statistically significant predictors of the WTP for improved solid waste management services. We checked the goodness of fitness using the Hosmer–Lemeshow goodness of fit.

### 2.9. Ethical Consideration

Institutional Health Research Ethical Review Committee of the College of Health and Medical Sciences, Dilla University, approved the protocol of the study (Ref. no. duchm/irb/038/2022). We obtained written informed consent from each study participant after explaining the purpose and benefits of the study. Each participant's collected personal information would be kept confidential and not shared.

## 3. Result

### 3.1. Sociodemographic Characteristics of the Respondents

Out of the 552 study participants, 414 (75%) were males, 496 (89.9%) were married, and 300 (54.3%) were in the age group of 30-45 years. The mean age of the respondents was 36 (SD = 8.44) years and ranged from 23 to 59 years. Out of the study participants, 40.9% had primary [1–8] education, 85% were Gedeo by ethnicity, 52.7% were orthodox religion followers, and 57.6% of them had a family size of less than or equal to five (Table 1).

### 3.2. Current Waste Practices and Household Participation

Out of the study participants, 511 (92.6%) reported that they had access to waste management services. Four hundred thirty-six (79%) of the study participants reported that they had no separate container for recyclable and nonrecyclable wastes. Almost more than half, 277 (53.9%), of the respondents were satisfied with the existing waste management service and 379 (68.7) of them generated 2 sacks of 50 kg of solid waste per week (Table 2).

### 3.3. Willingness to Join and Pay for Community-Based Health Insurance Scheme

**Figure 1:**
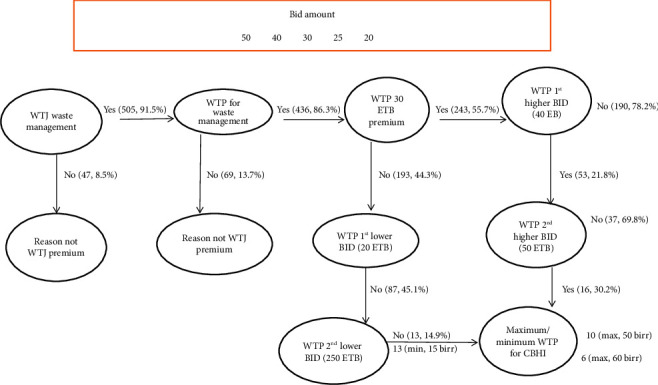
Participants' willingness to pay for solid waste management services, 2022.

**Figure 2:**
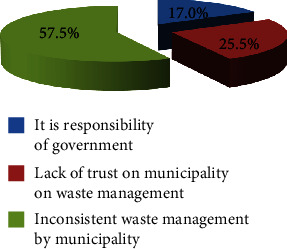
Participants' reason for not willing to join solid waste management services, 2022.

**Figure 3:**
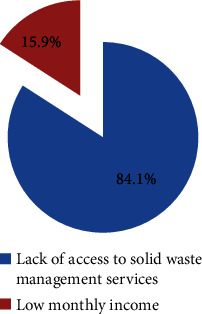
Participants' reason not willing to pay for improved solid waste management services, 2022.

Of the 552 study participants, 91.5% (95% CI: 89.2, 93.8) of them were willing to join in improving solid waste management services in their town (Figure 1). Of those who refused to join the services, 17% reported solid waste management as the responsibility of the government, 25.5% of them reported a lack of trust in the municipality in solid waste management, and 57.4% of them reported inconsistent solid waste collection and transportation by the municipality as the major reasons not to support solid waste management services (Figure 2). In addition, we found that 86.3% (95% CI: 83.3, 89.4) of respondents were willing to pay for improving solid waste management services in their town (Figure 1). The mean amounts of the contribution that participants were willing to pay were 28.37 (SD: 7.56) ETB or (0.55USD) per respondent per month. The study revealed that, among the 436 study respondents willing to pay, 243 (55.7%) were willing to pay the initial bid of 30 ETB (0.58USD), 53 (21.8%) of participants who were willing to pay the initial bid were willing to pay the first higher bid of 40 ETB (0.78USD), and 16 (30.2%) of respondents who were willing to pay the first higher bid were also willing to pay the second higher bid of 50 ETB (0.97USD). On other hand, one hundred ninety-three (44.3%) of the study participants were not willing to pay the initial contribution, with 87 (45.1%) of them were not willing to pay the first lower bid of 25 ETB (0.49USD) and 13 (14.9%) of respondents who were not willing to pay the first lowere bid were not willing to pay the second lower bid of 20 ETB (0.39USD). Out of 69 study participants who were not willing to pay for improving solid waste management services, 84.1% reported that the absence of access to solid waste management services was the main reason for not being willing to pay for improved solid waste management. In addition, 15.9% of respondents reason out that low monthly income was a factor for not being willing to pay for improving solid waste management services in their town (Figure 3).

### 3.4. Factors Associated with Respondents' Willingness to Join Waste Management

Variables such as age, sex, educational status, occupational status, family size, monthly income, house ownership, access to waste management services, amount of waste generated, households attitude, and knowledge about waste management were candidate variables for multivariate logistic regression (Table 3). In multivariate logistic regression, we found that educational status, family size, access to solid waste management services, amount of waste generated per week, households' attitudes, and knowledge about waste management showed statistically significant association with willingness to join waste management. We found that the odds of willingness to join for improving solid waste management practice were significantly higher among respondents with a family size greater than five (AOR = 3.68; 95% CI: 1.36–9.98) compared to their counterparts. Moreover, those who had primary education (AOR = 3.6; 95% CI: 1.45–8.97) and secondary and above education (AOR = 7.1; 95% CI: 1.93–26.18) were almost four and seven times more likely to support improving solid waste management services compared to their counterpart, respectively. Besides, we found the odd of willingness to join for improving solid waste management services was significantly higher among respondents who had a positive attitude (AOR = 4.23; 95% CI: 1.98–9.05) and good knowledge (AOR = 7.59; 95% CI: 3.21–17.97) of solid waste management as compared to those who had a negative attitude and poor knowledge of waste management services. Similarly, study participants who had access to solid waste management services were 3.02 times more likely to support improving solid waste management services compared to their counterparts (AOR = 3.02; 95% CI: 1.05–8.70). Moreover, the odds of willingness to join for improving solid waste management were almost 3 times higher among individuals who generated 2 and above sacks of 50 kg per week than those who generated 1 sack of 50 kg weekly (AOR = 2.99, 95% CI: 1.39–6.49) (Table 3).

### 3.5. Factors Associated with Respondents' Willingness to Pay for Waste Management

In the bivariate logistic regression, we considered variables with a *p* value < 0.25 as potential candidates for multivariate logistic regression analysis. Thus, we considered variables such as age, educational status, households' monthly income, access to waste management services, amount of waste generated per week, households attitude, and knowledge of solid waste management services for the multivariate logistic regression model. We found that study participants who had a monthly income of 1000-2000 ETB and greater than 2000 ETB were almost 4 times more likely to pay for improving solid waste management than those individuals who had a monthly income of less than 1000 ETB (AOR = 4.18, 95% CI: 2.21, 7.91 and AOR = 4.10, 95% CI: 1.87, 8.98, respectively). In addition, respondents who attended primary education and secondary and above education were 2.37 and 4.19 times more likely to pay for improving solid waste management services than those who had informal education (AOR = 2.37; 95% CI: 1.22–4.59 and AOR = 4.19; 95% CI: 1.79–9.80, respectively). Participants who had access to waste management services were 3.57 times more likely to pay for improving solid waste management services compared to their counterparts (AOR = 3.57, 95% CI: 1.36, 9.36). Study participants who generated 2 sacks of 50 kg of waste per week were 3.72 times more likely to pay for waste management services than those who generated 1 sack of 50 kg of waste weekly (AOR = 3.72, 95% CI: 2.10, 6.78). Furthermore, we found the odds willingness to pay for improving solid waste management services were significantly higher among respondents who had good knowledge (AOR = 3.58, 95% CI: 1.98, 6.48) of solid waste management as compared to those who had poor knowledge of waste management services (Table 4).

## 4. Discussion

Developing countries are facing challenges to manage solid waste generated due to economic growth, change in consumer behaviour, and lifestyles of people. Management of solid waste generated needs well designed and improve the existing waste management infrastructure through improved community participation [16]. Thus, this study assessed the willingness of households' in the Gedeo zone to join and pay for improving waste management services and its associated factors. This study found that 91.5% of households were willing to join for improving solid waste management services. The current finding is in line with a study from Addis Ababa, 91.02% [13], and higher than the study conducted in Nigeria, 80% [21], and Addis Ababa, Ethiopia, 76% [23]. The observed discrepancy might be due to differences in the study period and setting, level of awareness about the impact of solid waste on health, and access to solid waste management services. In this study, we found that study participants with large family sizes were more likely to join to improving solid waste management services than those with few family sizes. We found that the odd of willingness to join for improving solid waste management practice was significantly higher among respondents with a family size greater than five compared to their counterparts. This is explained by the respondents having high family size generating a high amount of solid wastes which have greater impact on the cleanness of their environment or compound [31]. Thus, households with high family members were more likely to support improving the existing solid waste management services in their town than those with few family sizes.

We observed that participants who had good knowledge of solid waste management services were more likely to support improving solid waste management services. In addition, we found that respondents who had a positive attitude about solid waste management were almost 4 times more likely to support solid waste management services than those who had a negative attitude. Furthermore, this study revealed that those study participants who attended primary and above were more likely to join improving solid waste management services than their counterparts. The possible explanations for these results could be that participants who were more educated have improved awareness and a better understanding of the impact of inappropriate solid waste management on their health, environment, and livestock in a distorted manner. Besides, respondents who had access to solid waste management services were almost 3 times more likely to support improving solid waste management services compared to their counterparts. This is explained by participants who had access to solid waste management services know the benefits of supporting improving the existing solid waste management services and might be more satisfied with the current services provided to them and willing to support solid waste management services. Furthermore, we found that the amount of waste generated weekly by respondents affects the willingness to support improving solid waste management services in their town. Individuals who generated a high amount of solid waste (2 bags of 50 kg per week) were more likely to support waste management services compared to those who generated less amount of solid waste weekly (1 bag of 50 kg per week). This could be because respondents who generated a high amount of waste need frequent waste collection and transportation to keep their environment or compound clean and are willing to support the comprehensive and well-managed solid waste management services than distorted solid waste management using daily laborer.

This study found that 86.3% of the study participants were willing to pay for improving solid waste management services. This is comparable with that of the study conducted in Macau, 85.4% [30]; Gondar, Ethiopia, 87.3% [32]; Bahir Dar, Ethiopia, 86.3% [24]; Injibara town, Ethiopia, 81.06% [25]; and Jimma town, Ethiopia, 83.5% [2]. However, this finding is higher than the study finding in Nepal, 61% [18]; Uganda, 48.1% [22]; and Tanzania, 63% [28]. The discrepancy might be due to differences in the socioeconomic status of communities, study period, community access to solid waste management services, and satisfaction with current solid waste management services. In addition, we found that the average amount of money that the respondents were willing to pay per individual per month was 28.37 ETB (0.55USD). This is lower than the study done in Nepal, 0.72USD [18]; Ghana, 1.74USD [33]; and Injibara town, Ethiopia, 29.7 ETB [25]. This finding is higher than the study finding in Bahir Dar, 13.1 ETB [24]; Mekelle, 11.89 ETB [19]; and Jimma, 17.26 ETB [2]. The discrepancy might be due to differences in the elicitation methods, initial bid, study period, and change in value of money over the time period.

In our study, participants' economic factor (i.e., monthly income) has a positive association with willingness to pay for improving solid waste management services. This result is supported by studies conducted in Malaysia [34], Nepal [18], Uganda [22], Zimbabwe [35], and Mekelle, Ethiopia [19]. This could be because participants who get a high monthly income have adequate money to pay for solid waste management services. In addition, the respondent who had higher economic status have bars, hotels, restaurants, and different enterprises that generate a huge amount of solid waste that affect the surrounding environments [31]. Thus, these respondents may support integrated solid waste management in their town to improve the cleanness of the surrounding environment and create a conducive place for their clients.

We found that participants who had good knowledge of solid waste management services were more likely willing to pay for improving solid waste management services than their counterparts. This finding is in line with that of studies done in Nepal [18], Vietnam [26], Ghana [5], and Mekelle, Ethiopia [19]. In addition, respondents who had better educational status were more likely to pay for improving solid waste management services. This is supported by findings from previous studies done in Malaysia [34, 36], Nepal [18, 37], Nigeria [21], Ghana [38], and Ethiopia [24]. This could be because an educated individual earns a high salary and can support the solid waste management services without financial problems. Besides, educated participants might easily understand the benefits of paying for improving solid waste management services in their town.

Respondents who generated 2 sacks of 50 kg of solid waste per week were more likely to pay for improving solid waste management compared to those who produced 1 sack of 50 kg of solid waste weekly. This finding is in line with previous studies that found individuals who generated huge amounts of solid waste were more likely to pay for improving solid waste management services [2, 24, 25]. In addition, we found that respondents who had access to solid waste management services were almost four times more likely to pay than those who had no access. This finding is in agreement with studies done in Bahir Dar city, Ethiopia [24], and Injibara town, Ethiopia [25], in which individuals who had access to solid waste management services were more likely to pay for improving solid waste management. This could be because individuals who can easily access solid waste management services were more satisfied with the service and were willing to pay to improve the current solid waste management services. Our study had several limitations. Firstly, we used the CVM to elicit WTP that does not indicate the actual amount of money the study participants want to pay based on their own choice. Secondly, the absence of previous studies on willingness to join for improved solid waste management challenged us to compare our findings. Lastly, we did not include qualitative studies in our studies, in which it is impossible to assess different factors that hinder communities to support improved solid waste management services.

## 5. Conclusion

This study found that a significant number of the respondents were willing to support and pay for improved solid waste management services. A higher probability of willingness to support and pay for solid waste management services among residents who had access to waste management services and good knowledge of solid waste management was observed. Therefore, wide-range awareness creation through mini media should be used to address poor knowledge. In addition, establishing more temporary waste collection sites in every corner of cities is needed to encourage households to support and pay for improved solid waste management services.

## Figures and Tables

**Table 1:** Sociodemographic characteristics of respondents in South Central Ethiopia, 2022.

Variables	Categories	Frequency (%)
Sex	Male	414 (75.0)
	Female	138 (25.0)
Age	18-29 years	168 (30.4)
	30-45 years	300 (54.3)
	46 and above years	84 (15.2)
Marital status	Married	496 (89.9)
	Others^a^	56 (10.1)
Religion	Muslim	106 (19.2)
	Orthodox	291 (52.7)
	Protestant	155 (28.1)
Educational status	Informal education	197 (35.7)
	Primary	226 (40.9)
	Secondary and above	129 (23.4)
Ethnicity	Gedeo	469 (85.0)
	Oromo	54 (9.8)
	Amhara	29 (5.3)
Occupation	Government employee	67 (12.1)
	Merchant	225 (40.8)
	Others^b^	260 (47.1)
Monthly income	<1000 ETB	142 (25.7)
	1000-2000 ETB	271 (49.1)
	Above 2000 ETB	139 (25.2)
House ownership	Yes	450 (81.5)
	No	102 (18.5)
Family size	≤5	318 (57.6)
	>5	234 (42.4)

^a^Widowed/divorced. ^b^Daily labor, farmer, and housewife. 1 USD = 51.54 ETB.

**Table 2:** Current waste management practices and household participation, 2022.

Variables	Categories	Frequency (%)
Amount of waste generated per week (in sack)	1 sack of 50 kg	173 (31.3)
	2 sacks of 50 kg	379 (68.7)
Distance of waste disposal	≤1 km	352 (63.8)
	>1 km	200 (36.2)
Presence of separate containers for recyclable and nonrecyclable wastes	Yes	116 (21.0)
	No	436 (79.0)
Had access to waste management service	Yes	511 (92.6)
	No	41 (7.4)
Perceived satisfaction with the current services	Very dissatisfied	15 (2.9)
	Not satisfied	141 (27.1)
	Neutral	81 (15.8)
	Satisfied	270 (52.5)
	Very satisfied	7 (1.4)

**Table 3:** Factors associated with respondents' willingness to join solid waste management services, 2022.

Categories	Willingness to join		Odds ratio(95%CI)	AOR(95%CI)
	Yes (%)	No (%)		
*Age categories*				
18-29 years	150 (27.2)	18(3.3)	1	1
30-45 years	227(41.1)	23(4.2)	1.45 (.756, 2.76)	1.15 (0.526, 2.50)
Above 45 years	78 (14.1)	6(1.1)	1.56(0.595, 4.09)	1.16(0.387, 3.47)
*Sex*				
Male	377(68.3)	37 (6.8)	1	1
female	128(23.2)	10(1.8)	1.26(0.607,2.60)	1.14 (0.520, 2.58)
*Educational status*				
Informal	171(31.0)	26(4.7)	1	1
Primary	215(38.9)	11(2.0)	2.97(1.43, 6.19)	3.6(1.45, 8.97)∗
Secondary and above	119(21.6)	10(1.8)	1.81(0.841, 3.89)	7.1(1.93, 26.18)∗
*Occupational status*				
Government employee	59(10.7)	8(1.4)	1	1
merchant	214(38.8)	11(2.0)	2.64(1.02,6.86)	2.7(0.624,11.19)
Other	232(42.0)	28(5.1)	1.12(0.487,2.59)	1.35(0.327,5.55)
*Monthly income*				
Less than 1000 birr	121(21.9)	21(3.8)	1	1
1000-2000 birr	253(45.9)	18(3.3)	2.44(1.26,4.75)	1.37(0.576,3.24)
Above 2000 birr	131(23.7)	8(1.4)	2.84(1.22,6.66)	1.24 (0.387,3.99)
*Access to waste management*				
No	30(5.4)	11(2.0)	1	1
Yes	475(8.6)	36(65.0)	4.84(2.24,10.44)	3.02(1.05,8.70)∗
*Family size*				
Less than or equal to 5	279(50.5)	39(7.1)	1	1
Greater than 5	226(41.0)	8(1.4)	3.95(1.81,8.62)	3.68 (1.36,9.98)∗
*House ownership*				
No	85(15.4)	17(3.1)	1	1
Yes	420(76.1)	30(5.4)	2.8(1.48,5.31)	1.69 (0.735, 3.89)
*Amount of waste generated per week*				
1 sacks of 50kg	143(25.9)	30(5.4)	1	1
2 sacks of 50kg	362(65.6)	17(3.1)	4.47(2.39,8.35)	2.99(1.39,6.49)∗
*Overall Attitude*				
Negative	110(19.9)	25(4.5)	1	1
Positive	395(71.6)	22(4.0)	4.1(2.22,7.52)	4.23(1.98,9.05)∗
*Overall Knowledge*				
Poor knowledge	163(29.5)	38(68.9)	1	1
Good Knowledge	342(62.0)	9(1.6)	8.86(4.19,18.76)	7.59(3.21,17.97)∗

Note: ^∗^*p* value < 0.05.

**Table 4:** Factors associated with respondents' willingness to pay for improved solid waste management, 2022.

Categories	Willingness to pay		Odds ratio(95%CI)	AOR(95%CI)
	Yes (%)	No (%)		
*Age categories*				
18-29 years	125 (24.8)	25(5.0)	1	1
30-45 years	240 (47.5)	37 (7.3)	1.3 (.747, 2.25)	1.29 (.694, 2.38)
Above 45 years	71 (14.1)	7(1.4)	2.03(0.835, 4.93)	2.03(0.764, 5.41)
*Educational status*				
Informal	135 (26.7)	36(7.1)	1	1
Primary (1-8)	193(38.2)	22(4.4)	2.34(1.32, 4.16)	2.37(1.22, 4.59)∗
Secondary and above	108(21.4)	11(2.2)	2.62(1.27, 5.39)	4.19(1.79, 9.80)∗
*Monthly income*				
Less than 1000 birr	86 (17.0)	35(6.9)	1	1
1000-2000 birr	230(45.5)	23(4.6)	4.1(2.28,7.28)	4.18(2.21,7.91)∗
Above 2000 birr	120(23.8)	11(2.2)	4.5(2.14,9.23)	4.10(1.87,8.98)∗
*Access to waste management*				
No	18(3.5)	12 (2.4)	1	1
Yes	418 (82.8)	57 (11.3)	4.89(2.24,10.68)	3.57(1.36,9.36)∗
*Amount of waste generated per week*				
1 sack of 50kg	105(20.8)	38(7.5)	1	1
2 sacks of 50kg	331(65.6)	31(6.1)	3.87(2.29,6.52)	3.72(2.10,6.78)∗
*Overall Attitude*				
Negative	92(18.2)	18(3.6)	1	1
Positive	344(68.1)	51(10.1)	1.32(0.736,2.37)	1.53(0.777,3.02)
*Overall knowledge*				
Poor knowledge	122(24.2)	41(8.1)	1	1
Good knowledge	314(62.2)	28(5.5)	3.77(2.23,6.37)	3.58 (1.98, 6.48)∗

Note: ^∗^*p* value < 0.05.

## Data Availability

The datasets used or analyzed during this study were available from the corresponding author on reasonable request.

## Abbreviations

AOR: Adjusted odds ratio
CI: Confidence interval
COR: Crude odds ratio
CVM: Contingent valuation method
ETB: Ethiopian birr
HEPI: Health Professionals Education Partnership Initiatives
IRB: Institutional Review Board
LMIC: Low- and medium-income countries
OR: Odds ratio
SWM: Solid waste management
USD: United State Dollar
WHO: World Health Organization
WTP: Willingness to pay.
